# The Role of Apoptotic Signaling in Axon Guidance

**DOI:** 10.3390/jdb6040024

**Published:** 2018-10-18

**Authors:** Riley Kellermeyer, Leah M. Heydman, Grant S. Mastick, Thomas Kidd

**Affiliations:** Department of Biology/MS 314, University of Nevada, 1664 N. Virginia St., Reno, NV 89557, USA; rileykellermeyer@gmail.com (R.K.); leah.heydman@gmail.com (L.M.H.); gmastick@unr.edu (G.S.M.)

**Keywords:** axon guidance, growth cone, cytoskeleton, caspases, apoptosis, signal integration, basal level of caspase activity, death associated inhibitor of apoptosis, axon branching, Netrin, DCC, Frazzled, Slit, Robo, *Drosophila*

## Abstract

Navigating growth cones are exposed to multiple signals simultaneously and have to integrate competing cues into a coherent navigational response. Integration of guidance cues is traditionally thought to occur at the level of cytoskeletal dynamics. *Drosophila* studies indicate that cells exhibit a low level of continuous caspase protease activation, and that axon guidance cues can activate or suppress caspase activity. We base a model for axon guidance on these observations. By analogy with other systems in which caspase signaling has non-apoptotic functions, we propose that caspase signaling can either reinforce repulsion or negate attraction in response to external guidance cues by cleaving cytoskeletal proteins. Over the course of an entire trajectory, incorrectly navigating axons may pass the threshold for apoptosis and be eliminated, whereas axons making correct decisions will survive. These observations would also explain why neurotrophic factors can act as axon guidance cues and why axon guidance systems such as Slit/Robo signaling may act as tumor suppressors in cancer.

## 1. Introduction

The navigational center of growing axons is the growth cone, a highly dynamic expansion of the axon shaft that samples the environment and integrates multiple cues to generate directed extension, retraction, and turning [1,2]. Traditionally, axon attractants such as Netrins are thought to increase cytoskeletal outgrowth towards a cue, whereas axon repellents such as Slits inhibit cytoskeletal growth [3,4]. The net effect of attractive and repulsive cues on the cytoskeleton results in growth towards attractive cues and away from repulsive cues. Integration has been demonstrated to also occur through interactions between cell surface receptors, and through intracellular kinases [5,6]. More recent evidence suggests that the traditional view of growth cones navigating up or down gradients of guidance cues such as Netrin may not be accurate or even valid in vivo [7,8,9,10,11]. Our own work led us to identify a role for the apoptotic machinery in the growth cone that is likely functioning to integrate opposing guidance cues. This review examines models for how the cell death machinery could be involved in axon guidance with an emphasis on results from *Drosophila*.

### Drosophila Netrin-B Is a Neurotrophic Factor That Blocks Cell Death

Netrins are diffusible axon guidance cues most famous for attracting axons to the CNS midline [12]. The fly has two Netrin genes, *NetA* and *NetB*, that are required for midline and motor neuron axon guidance [13,14]. The genes appear to be the product of tandem duplication and display a high degree of functional overlap. Netrins are expressed by the CNS midline and embryos lacking both Netrins (*NetAB*) have axon guidance defects and increased cell death. Both phenotypes can be rescued by midline expression of either gene. Localized sources of Netrins therefore appear to provide a navigational cue. In contrast to midline expression, pan-neuronal expression of either Netrin in wild type embryos leads to axon phenotypes, either due to a lack of positional information specific to the midline or through attraction to non-midline areas [13,14]. It was therefore surprising to find that pan-neuronal expression of *NetB* alone can rescue axon guidance defects in *NetAB* embryos [15]. In contrast, pan-neural expression of *NetA* increases the severity of the *NetAB* mutant phenotype, establishing a clear difference between the proteins. NetB was subsequently identified as a neurotrophic factor when over-expressed, because blocking cell death using the baculovirus p35 caspase inhibitor in discrete subsets of neurons can rescue *NetAB* midline guidance defects [15]. These findings substantiate a model for a non-apoptotic role for caspases in the growth cone, as suggested by prior results in Xenopus retinal growth cones and other systems [16,17,18]. 

## 2. The Apoptotic Machinery and Guidance Receptors

**Figure 1:**
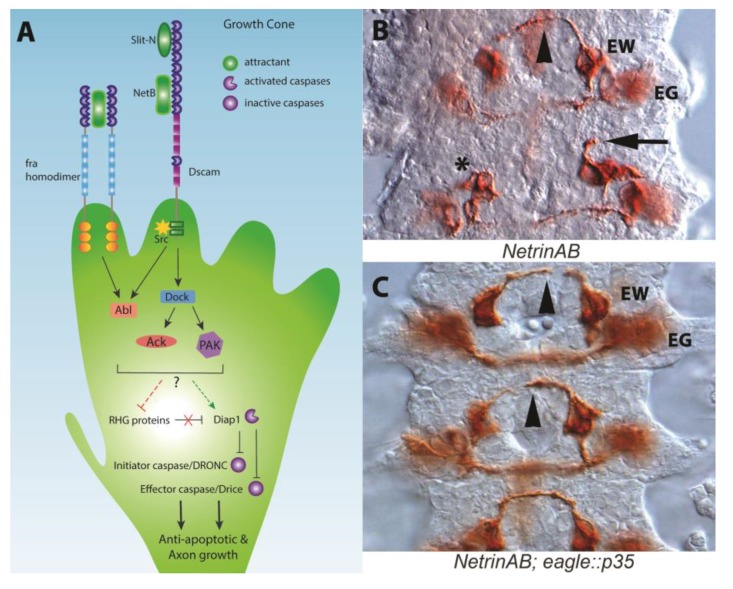
Apoptotic machinery and axons. (**A**) Schematic of axon guidance signaling pathways that potentially interact with the apoptotic machinery. Candidate downstream effectors are shown based on proteins that can promote survival and are known to interact with the receptors shown in *Drosophila* [19,20,21,22,23,24]. Cytoplasmic signaling components may act on caspase regulators, such as inactivating RHG proteins, promoting Diap1 function or could act directly on the caspases (uncertainty indicated by dotted lines). (**B**) A *Drosophila* embryo lacking the *Netrin-A* and *Netrin-B* axon guidance genes (*NetAB*) stained to reveal *eagle* positive axons (brown). Anterior is to the top. The EW and EG neuron clusters are indicated. In *NetAB* mutants, the EW commissure successfully crosses the midline about 50% of the time (arrowhead). A growth cone can be seen remaining projecting ipsilaterally (arrow). An EW cluster lacking a leading growth cone either due to developmental delay or apoptosis of a neuron can also be seen (asterisk). An overall lack of symmetry of the EW and EG cluster can be seen with the clusters mispositioned and differences in neuron number reflected in differences in staining intensity. The EG commissures are present but not in the plane of focus. (**C**) A *Drosophila NetAB* embryo whose axon guidance defects have been rescued by expression of the *p35* effector caspase inhibitor [25,26]. Growth cones of the EW neuron cluster can be seen crossing the midline (arrowheads), while the growth cones of the contralateral homologues are growing at a slower rate. The growth cones of the most posterior segment in this panel have fasciculated with the contralateral homologue even though the more anterior segments are slightly older in development. In older embryos 90% of the EW axons cross the midline. Images courtesy of G. Newquist.

Programmed cell death or apoptosis is a key part in the development of multicellular organisms and in the maintenance of the correct number of cells in mature animals [27]. The classic role for cell death in neural development is to eliminate unneeded connections that fail to compete for a limiting survival factor, the neurotrophic hypothesis [28]. For example, axons that are misguided and fail to reach their target tissue die [29]. Further research has shown that neurotrophic factors can also induce death in certain contexts, and this activity is functionally conserved in the fly [30,31,32]. Apoptosis operates through molecular cascades, offering several potential signaling nodes that could intersect with axon guidance signaling pathways. A crucial event in apoptosis is the activation of specialized cysteine-aspartic acid proteases called caspases (Figure 1A). Signals derived from the mitochondria, in response to external pro-apoptotic signals or the withdrawal of trophic support molecules, trigger activation of initiator caspases [33,34,35]. Activation relies on multimerization and/or conformational changes of initiator caspases, like Dronc, leading to proteolytic activation of effector caspases such as Drice and Dcp-1. This amplification of effector caspases is then responsible for the extensive proteolytic cleavage that occurs during apoptosis. Caspase activity is buffered by the death-associated inhibitor of apoptosis protein family (*Diap1*/*thread*), the viral p35 protein and other specialized inhibitors which oppose caspase activation [36,37,38,39,40,41]. Many caspase inhibitors, like Diap1, are continuously active to prevent apoptosis by marking initiator caspases for degradation [42,43]. To initiate apoptosis, Diap1 is cleaved by inhibitor of apoptosis antagonists, notably Hid, Grim and Reaper (RHG proteins), ultimately allowing caspase activation [34]. In addition to apoptosis, caspases have been implicated in actin dynamics of *Drosophila* spermatid individualization [44,45], sense organ precursor selection [42,46], and dendrite retraction [47,48] (reviewed in [35]). Together, this cascade of apoptotic regulators is present in all cells at low levels, but normally kept in check by specific and tightly regulated modulators. 

The apoptotic machinery is present in the growth cone of extending axons and can be activated by external signals such as Netrin [16]. The principal vertebrate Netrin receptor is DCC (Deleted in Colorectal Cancer; Frazzled in the fly), which dimerizes upon ligand binding to stimulate signaling pathways that alter cytoskeletal dynamics [12]. Netrins are also capable of repelling axons through the Unc-5 receptor [49]. In vertebrates, failure of DCC to homodimerize triggers cell death via the initiator caspase-9 and the effector caspase-3 [50]. The dual function of DCC to transduce both migratory and apoptotic signals is known as the dependence receptor hypothesis, wherein cell survival is dependent on ligand occupancy of receptors. Dependence receptors such as DCC are characterized by having caspase cleavage sites in their cytoplasmic domains and that the absence of the ligand triggers caspase cleavage of the receptor. The DCC caspase cleavage site is required to trigger apoptosis, and mutation of the site in mice prevents tumor suppression [51,52]. However, the ability of Netrin to act as a survival factor, particularly in spinal cord development is controversial as conflicting survival phenotypes have been observed by different groups [53,54]. Additional recent evidence strongly suggests that the dependence receptor mechanism is not operating in the mouse spinal cord [55]. 

In flies, the DCC homologue, *frazzled* (*fra*; also called Unc-40 in *C. elegans*), lacks the caspase cleavage site in the cytoplasmic domain, suggesting Fra is not a dependence receptor, though alternative sites could exist [54,56]. Additionally, the **loss** of *fra* activity triggers apoptosis in some tissues, rather than being protective from cell death, as would be expected from the loss of a dependence receptor [57]. The ability to rescue the axon guidance defects of *NetAB* mutants by blocking apoptotic signaling therefore requires an alternative explanation to the dependence receptor hypothesis.

## 3. How Could Caspase Signaling Operate in Growth Cone Guidance?

Apoptosis requires major changes in the structure of cells via rearrangements of the cytoskeleton, so it is not surprising that caspases cleave a large number of cytoskeletal and structural proteins such as actin, alpha-tubulin, and Spectrin [58,59,60,61]. Caspase-3 cleaves Spectrin in growth cones in culture [62]. Changes in levels and localization of cytoskeletal proteins are observed during apoptosis of larval salivary glands, and these changes are prevented by inhibiting caspases [63]. Many proteins that modulate the cytoskeleton are also cleaved during apoptosis such as cofilin, GAP43, and rho kinase (Rock), as well as many cell adhesion molecules [64,65,66]. These molecules could serve as caspase substrates in the growth cone and their cleavage would likely decrease protrusion (actin driven extension of filopodia and lamellipodia) and inhibit axon outgrowth. Inhibiting caspase activity would protect cytoskeletal components from proteolysis and potentially increase protrusion and forward movement. Migrating border cells in the *Drosophila* ovary provide an apoptosis-independent example for this model. Normal border cell migration requires the activity of the *Diap1* caspase inhibitor [67]. Diap1 forms a complex with the actin cytoskeletal modulators Rac and Profilin, and Diap1 protects these modulators from degradation by Dronc. In ovary border cell migration, Diap1 alters cytoskeletal dynamics independent of apoptosis inhibition, as loss of Diap1 in these cells does not result in cell death and migration phenotypes are not rescued with p35. Similarly, Diap1 promotes F-actin assembly in polarized elongation of sensory organ progenitors by blocking Dronc activation, in a caspase-dependent, apoptosis-independent manner [68,69]. F-actin turnover at the cell margin therefore requires inhibition of Diap1 without any effect on cell survival. An additional example of non-apoptotic functions of caspases comes from the study of so-called “undead” cells in which Hid expression activates apoptosis, but the cells are kept alive by co-expression of p35 [70]. When these cells are created in the anterior‒posterior boundary of the wing disc, the undead cells invade and migrate into the posterior domain of the wing disc. This migration is dependent on effector caspases and appears to require intermediate levels of caspase activity [71,72]. Comparable approaches have been taken in vertebrate neurons to separate axon growth from neuronal survival. Blocking cell death through over-expression of Bcl-2 or loss of Bax function was used to demonstrate that axon growth does not appear to be constitutive but likely relies on external signals [73,74]. 

There are several important implications from these and other studies for non-apoptotic functions of caspase protease activity. The first is that activation of the apoptotic machinery does not necessarily lead to cell death. In vertebrates, molecular mechanisms have been identified that prevent complete activation of the apoptotic cascade [75]. Studies of the synapse suggest that transient and local activation of caspases can remodel the synapse [76], and observations of activated caspase-3 restricted to the growth cone and sites of axon branching are consistent with this model [16,77]. Second, *Drosophila* studies have demonstrated that there is a continuous low level of initiator caspase activation through auto-processing and the role of Diap1 is to counteract this basal caspase activity [43,78,79,80,81]. Low levels of caspase activation have been visualized in wing discs using the FRET-based SCAT3 caspase activity probe [42]. A vertebrate Diap1 homologue, X-linked inhibitor of apoptosis (XIAP) plays a role in limiting caspase activation [82,83], and appears central to restricting caspase activation to subcellular compartments of neurons [84]. 

**Figure 2:**
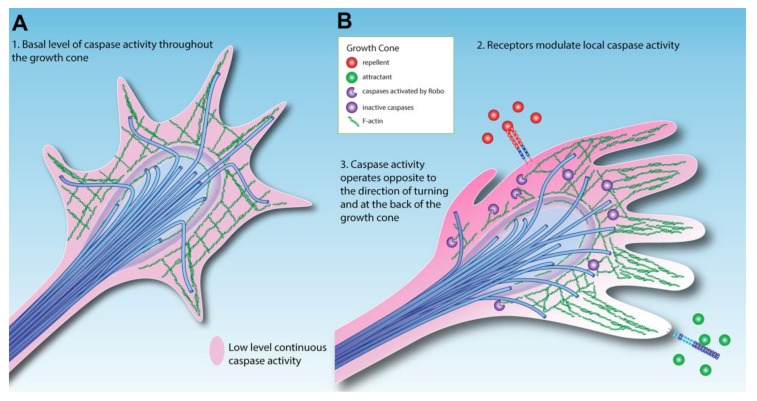
Model for caspase activity in the growth cone. (**A**) Simple low-level activation of caspases throughout the growth cone. (**B**) Attractant and repellent cues modulate caspase activity via cell surface receptors, altering the growth cone trajectory. In this model, repellents increase caspase activity, while attractants decrease caspase activity.

Returning to the fly CNS and the observation that Netrin mutant phenotypes can be rescued by anti-apoptotic factors, the simplest model to explain the effects of caspase inhibition in *Netrin* mutants is that there is a basal level of caspase activation in growth cones that has to be overcome for maximal forward growth (Figure 2A). This model was first proposed by Gilman and Mattson, after demonstrating that addition of caspase inhibitors to neuronal cultures increases axon outgrowth [76]. The implication is that a basal level of caspase proteolysis keeps normal axon growth below maximum levels, although the molecular targets of caspases in growth cones remain undefined. To support any of the models described, cleavage of specific substrates, such as cytoskeletal components, will have to be demonstrated in the growth cone itself, which may require the study of larger growth cones from other invertebrate or vertebrate species, given the small size of *Drosophila* growth cones. 

It is worth noting that caspases can also modulate the actin cytoskeleton independently of their protease roles, such as promoting Aip1/cofilin mediated actin polymerization in migrating lymphocytes, though it is not known how widespread this effect is [65]. 

## 4. Extracellular Modulation of Caspase Activity in the Growth Cone

An exciting possibility arising from this simple model of caspase-mediated guidance is that caspase activity could be actively modulated in response to external cues (Figure 2B). Mehlen has proposed that axon guidance signaling pathways actively modulate tumor cell survival, explaining why axon guidance molecules are implicated in preventing cancer [85]. Despite a large number of studies implicating Slit/Robo signaling as tumor suppressors in cancer, a direct link of Slit/Robo signals to apoptosis has not yet been shown [86]. In fly screens, the post-translational regulator of Robo, *commissureless* and its paralogue *comm3*, have been shown to suppress apoptotic phenotypes through unidentified mechanisms [87]. DCC has been strongly linked to caspase activity [50], and DCC acts as a tumor suppressor via its dependence receptor activity [52]. In flies, the Netrin receptor *frazzled* appears to act as a tumor suppressor, because while *fra* mutant clones are usually not viable, they can be rescued with p35 expression blocking cell death [57]. Additionally, loss of *fra* activity leads to invasive cell phenotypes reminiscent of metastasis [57,88]. Mutant clones of *fra* have not been made in the embryo, but *fra* homozygotes do not display increased cell death in the developing CNS [15]. This could reflect a tissue-specific difference between the embryo and eye-antennal discs, perhaps due to functional overlap between survival factors in the embryo. Alternatively, the differences between homozygous embryos and mutant clones could be the result of cell competition in which mutant clones are less fit than neighboring cells, a process that interestingly can involve Slit/Robo signaling [89]. In the embryo, NetB likely promotes axon growth by inhibiting caspase activity in the growth cone. Double mutants for the *fra* and *Dscam1* Netrin receptors display an increase in cell death, whereas there is no change in either mutant alone [15], suggesting multiple receptors may mediate this activity.

**Figure 3:**
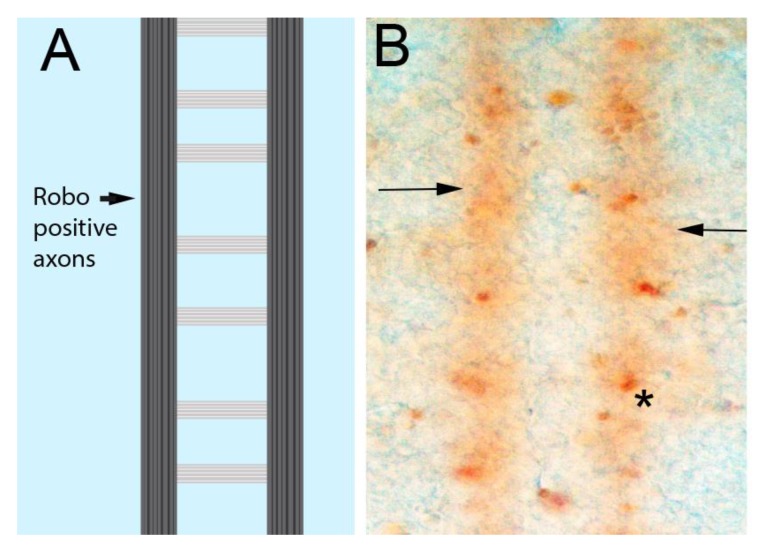
Low-level caspase activity in longitudinal axons. (**A**) Schematic of the CNS axon scaffold in the fly ventral nerve cord. The axons form a ladder-like pattern. The Robo repulsive receptor is only found in the longitudinal portions of CNS axons (arrow). (**B**) *Drosophila* embryonic nerve cord stained with an antibody raised against activated vertebrate Caspase-3 that appears to detect Dronc activation in flies [101]. Dying cells are visible as densely stained regions, usually oval in shape (asterisk). A continuous low level of staining can be seen in the region occupied by the longitudinal axons (arrows). This pattern matches the pattern of Robo localization, with the Robo protein excluded from axons segments crossing the midline while upregulated in axons using active Slit/Robo signaling to avoid the midline [102], suggesting that the Robo and caspase activation pathways may be linked.

Emerging evidence reveals that repellent signaling pathways are able to activate caspases, in some cases through direct binding. The Slit/Robo, Eph/Ephrin and Sema/Plexin pathways all recruit and/or activate caspases [17,77,90,91,92,93,94]. Slit/Robo signaling in zebrafish axons has been shown to genetically interact with caspases, in a manner that suggests localized activation [77]. Consistent with Slit/Robo regulation of caspases, we have observed low levels of activated caspase in a pattern that matches Robo localization in the ventral nerve cord (Figure 3). This suggests that caspase activity in the growth cone is increased by axon repellents and decreased by attractants, allowing axon outgrowth in the direction of attractant cues (Figure 2B). *Drosophila* motor neurons similarly integrate information concerning the levels of attractants and repellents emanating from their target muscle [95]. Thus caspase activation and inhibition could sum the input signals to determine lowered caspase activity domains where forward growth occurs, while a widespread basal level of inhibitors like Diap1 restrict the spread of local caspase activation by repellents. In this respect, the model resembles the synapse where the duration and intensity of caspase activation determines the difference between synaptic remodeling and neuronal death [96,97].

Dendritic pruning also requires localized caspase activation but over a much larger subcellular area (dendritic trees can be quite extensive compared to a growth cone), ultimately leading to the degradation of dendrites. Pruning relies heavily on the activation of initiator caspases such as Dronc [47,48]. Expression of p35, which blocks effector caspases, is either ineffective at blocking pruning [47], or only appears to delay pruning [98]. Migration of undead cells also requires initiator but not effector caspase activity, as migration is blocked when Diap1 is expressed (Diap1 inhibits both initiator and effector caspases) [71]. A distinguishing feature of non-apoptotic caspase activity may be a reliance on initiator caspases. Blocking cell death upstream of all caspases in fly embryos has remarkably little effect on the CNS axon scaffold [99], suggesting that caspase activity is dispensable for normal development. However, in sensitized backgrounds such as *NetAB* mutants, blocking effector caspases is sufficient to rescue axon guidance [15]. Overall these results suggest that there may be quantitatively different levels of caspase activation. Growth cones and migrating cells may display low continuous levels of caspase activation. Higher levels may be required for neuronal remodeling events in which structures or parts of the cell such as dendrites are lost, and the highest levels will of course lead to apoptosis. A mechanism for different levels of caspase activation has been proposed from observations of dendritic pruning. The model proposes that there is a threshold for cell death and that, below this threshold, IAPs bound to caspases can be quickly released without activation of the apoptosome [100]. This may represent a general mechanism for non-apoptotic functions of caspases but may need further refinement to account for multiple levels of caspase activation.

If axon repellents activate caspases at low levels, they could mediate contact inhibition or increase sensitivity to survival factors in tumors. Alternatively, it could be that cytoskeletal rearrangements in response to both positive and negative cues require caspase activation, or that caspase activation controls growth cone protein levels [16]. 

## 5. Caspase Signaling at the Fly CNS Midline

In fly *NetAB* mutants, most growth cones orient towards the CNS midline but many fail to cross it [103]. The original interpretation of this observation is that Netrins are required for axons to extend across the midline, rather than to attract axons to the midline. The ability of caspase inhibition to rescue axon crossing in *NetAB* mutants suggests that caspase activity inhibits midline crossing or axon outgrowth in general. To explain this observation, we propose that there is a low level of caspase activity in the growth cone that needs to be overcome for forward growth. Alternatively, or in parallel, strict temporal control of caspase activation may allow for the necessary cytoskeletal rearrangements [35]. While many axons do not, a significant number of axons do cross the midline in *NetAB* mutants, revealing that there are undiscovered attractants expressed at the midline. This suggests that caspase inhibition may increase axon outgrowth enough to allow growth cones to use these other cues to locate and grow towards the midline. Interestingly, the fly CNS midline is a source of other neurotrophic factors like the *Drosophila* neurotrophins DNTs [104], acting through neuronal Toll receptors [31]. Classically, the axonal target tissue produces these neurotrophic factors. An increasing number of examples are now known where intermediate targets supply neurotrophic factors—a phenomenon termed *en passant* or pre-target neurotrophic action [54,105,106,107]. As *NetB* and *DNTs* are expressed by the CNS midline intermediate targets, and required for motor axon targeting [95,108], both gene families may function as *en passant* neurotrophic factors and as guidance cues. Interestingly, midline glia require the axon derived epidermal growth factor Spitz to survive, and when the axons fail to contact the midline glia in *commissureless* mutants, the midline glia migrate to the axons to obtain Spitz [109]. Artificially promoting survival by increasing MAPK signaling, which inhibits Hid, removes the necessity for migration suggesting that Spitz is acting as a caspase-mediated attractive signal. Searching for survival factors could be a general mechanism that influences cell and growth cone migration. MAPK signaling in vertebrate growth cones and commissural axons has been implicated in the response to Netrin supporting this model [16,50]. Neurotrophic factors appear to be required for axon growth independent of their role in cell survival [110], and could do so by inhibiting caspase activation in the growth cone. Together, *Drosophila* axon navigation across the ventral midline suggests that there remains much to be discovered about the functional links between the classical axon guidance problem of midline crossing and caspase signaling.

## 6. Apoptotic Signaling in Axon Branching

As axons extend, particularly as they enter their target tissues, they also branch, with each branch forming its own growth cone. The process of axon branching is also likely regulated by the apoptotic machinery. One of the most dramatic visualizations of caspase activity in axons is in zebrafish retinal ganglion cells [77]. Caspase activation occurs in a dynamic fashion at branchpoints in developing axonal arbors and genetically interacts with Slit/Robo signaling. Interestingly, Slit is proteolytically cleaved into two fragments, Slit-N and Slit-C. Slit-N stimulates axon branching, whereas full length Slit (Slit-FL) inhibits branching [111,112,113]. Slit-N is neurotrophic [114], and it will be interesting to see whether Slit-FL can directly activate caspases, perhaps via p38 MAPK signaling as suggesting by zebrafish studies [77]. Additionally, activated caspase activity has been observed in the developing auditory brainstem within several segments of navigating axons as well as their terminal branches within targets where it is proposed to limit unnecessary axonal arborization, because inhibition of caspase activity causes axon branches to spread into inappropriate target tissue [18]. Although much less is known about axon branching within targets, caspase activity appears to be an important regulator of not only primary axons but also their terminal branches. Axon branching may be more important than primary axon growth for regenerative recovery of connections after injury or disease, as branching from spared axons can be major contributors to restoring circuit function [115]. 

## 7. Critical Experiments for the Activated Caspase Model

The most important step forward for the proposed model would be to observe asymmetric activation of caspases in growth cones exposed to attractants or repellents. The long axons of zebrafish retinal ganglion cells have been used to successfully demonstrate localized caspase activity using the SCAT3 probe [77]. It seems likely that the same approach could be used in the growth cones of cultured neurons, as asymmetric gradients of phosphorylated Shootin1 (a cytoplasmic axon outgrowth protein) have been observed in growth cones responding to shallow Netrin gradients [116]. Antibodies against activated (cleaved) caspases do not appear to have sufficient sensitivity at present. If Diap1 levels are modulated in response to guidance cues, as seems likely, using split fluorescent protein strategies to tag the endogenous protein and limiting fluorescence to small subsets of neurons may prove invaluable for in vivo analysis [117]. Both cell culture and in vivo approaches would be especially powerful if combined with high-resolution structured illumination microscopy, as has been used for dendritogenesis in *Drosophila* embryos [118]. A second important step for the model would be to define which components of the apoptotic machinery are used in axon guidance and to demonstrate genetic interactions with specific signaling pathways, as has been done for axon degeneration and pruning [119]. A technical problem will be to limit cell death in mutant combinations as this could greatly hinder the interpretation of phenotypes. Dose-sensitive genetic interactions will likely allow sensitization of the cell death machinery without triggering apoptosis. For example, *Diap1* heterozygotes would be predicted to increase caspase activity and enhance *NetAB* axon phenotypes without preventing analysis of axon guidance. Positive results in the proposed experiments would establish the basic model. We also propose that accumulating levels of caspase activation by axon errors will eventually reach the threshold to trigger apoptosis. This model requires an integration system to add up the errors over time, with the simplest mechanism being the accumulation of active caspases. Demonstrating that caspase activation persists and accumulates would require in vivo imaging. The Apoliner caspase reporter in which caspase cleavage results in nuclear translocation of enhanced green fluorescent protein (eGFP) while leaving monomeric red fluorescent protein (mRFP) at the cell membrane could function in this approach [120]. For example, mis-navigating *eagle* commissural axons in *NetAB* mutants would be predicted to have higher levels of eGFP in the nucleus than those that had successfully crossed the midline. A simpler and potentially complementary approach not requiring live imaging would be to use the CD8::Parp::Venus reporter to demonstrate higher levels of caspase activation in the axons of mis-navigating axons [48]. Additional reporters of caspase activity could similarly be employed [121].

## 8. Conclusions

The neurotrophic hypothesis proposes that competition between neurons for functional connections leads to the correct wiring of the nervous system. Studies of caspases now suggest that the apoptotic machinery plays an active role in forming the connections in the first place. Based on *Drosophila* studies, we propose that there is a continuous low level of basal caspase activation in growth cones that is kept in check by the caspase inhibitor Diap1. Modulation of caspase activity by external signals affects axon growth rates and allows for the integration of multiple, potentially conflicting, inputs to generate a coherent response. These conclusions are summarized:

Highlights of apoptotic signaling in axon guidance

- Localized activation of caspases in the growth cone may modulate axon guidance.
- Axon attractants can promote cell survival, while repellents can promote cell death.
- Neurotrophic factor effects on axon guidance could be through caspase signaling.
- Based on an analogy with systems in which caspase signaling has non-apoptotic roles, we propose that the duration and intensity of caspase activation can modulate growth cone activity, while longer and stronger caspase activity can induce death.
- Crossing the CNS midline is associated with lower caspase activity.
- Correct wiring of the nervous system could result from the elimination of incorrectly navigating neurons due to increased activity of the cell death machinery.
